# A Prospective Observational Registry of Repository Corticotropin Injection (Acthar® Gel) for the Treatment of Multiple Sclerosis Relapse

**DOI:** 10.3389/fneur.2020.598496

**Published:** 2020-12-22

**Authors:** Jeffrey Kaplan, Tamara Miller, Matthew Baker, Bryan Due, Enxu Zhao

**Affiliations:** ^1^Kansas City Multiple Sclerosis and Headache Center, Overland Park, KS, United States; ^2^Advanced Neurology of Colorado, LLC, Fort Collins, CO, United States; ^3^Collier Neurologic Specialists, LLC, Naples, FL, United States; ^4^Mallinckrodt Pharmaceuticals, Bedminster, NJ, United States

**Keywords:** repository corticotropin injection (RCI), Acthar Gel, multiple sclerosis, registry trial, exacerbation, relapse

## Abstract

**Background and Purpose:** Effective relapse treatment is critical for minimizing disability in patients with multiple sclerosis (MS). Repository corticotropin injection (RCI; Acthar® Gel) has demonstrated efficacy for the treatment of MS exacerbations. However, there is limited real-world evidence available regarding the relationship between the use of RCI for MS relapses and patient demographics, disease characteristics, and dosing regimens. In this multicenter, prospective, observational registry, patients receiving RCI for acute MS relapse were characterized, and recovery and safety outcomes were described.

**Methods:** Patients were invited by their treating clinician to participate in the registry during a routine care visit. The decision to initiate RCI occurred before determination of study eligibility. All treatment decisions were made at the discretion of the patient's health care provider and were not mandated by the study design or protocol. Each enrolled patient was followed for up to 24 Months or until the date of study termination. The primary endpoint was the change from baseline in MS Impact Scale Version 1 (MSIS-29v1) physical subscale scores at Month 2. Additional assessments included the MSIS-29v1 psychological subscale, Expanded Disability Status Scale (EDSS), Clinical Global Impression of Improvement (CGI-I), Work Productivity and Activity Impairment Questionnaire: MS (WPAI:MS), and Health Resource Utilization (HRU) questionnaire.

**Results:** Of 145 patients enrolled, 82 (56.6%) completed 24 Months of follow-up. Mean MSIS-29v1 physical subscale scores improved at 2 Months (−8.0; *P* = 0.0002) and 6 Months (−9.6; *P* < 0.0001). Mean MSIS-29v1 psychological subscale scores also improved at 2 Months (−7.9; *P* = 0.0040) and 6 Months (−9.9; *P* = 0.0012). Mean EDSS scores improved at 2 Months (−0.4; *P* < 0.0001) and 6 Months (−0.5; *P* < 0.0001). CGI-I scores indicated improvement in 63.4% of 71 patients at 2 Months and 61.4% of 57 patients at 6 Months (both *P* < 0.0001). Improvements on the WPAI:MS activity impairment domain (*P* < 0.001) and reductions in outpatient, specialist, and emergency department visits were observed at 2 and 6 Months. A total of 35 (28.0%) patients reported 83 adverse events; 11 (8.8%) patients reported 16 serious adverse events.

**Conclusions:** This observational study found significant improvements in MS assessment scores after RCI treatment and supports the efficacy and tolerability of RCI for MS relapse.

**Clinical Trial Registration:** This trial is registered on ClinicalTrials.gov with the identifier NCT02633033.

## Introduction

The most common form of multiple sclerosis (MS) is relapsing-remitting MS (RRMS), where patients experience relapses with episodes of acute neurologic dysfunction followed by partial or complete recovery periods (1, 2). Relapses in MS patients are associated with impaired daily abilities, residual disability, and reduced quality of life (1, 2).

Despite advances in the treatment of RRMS, patients continue to experience relapses. Several new drugs have been developed for MS treatment, but corticosteroids (typically methylprednisolone or prednisone) are the primary treatment for relapses (3). Effective relapse treatment is critical for minimizing duration of acute disability (4), but some patients do not respond to corticosteroid treatment or experience adverse effects and thus require an alternative treatment (1).

Repository corticotropin injection (RCI; Acthar® Gel) is approved by the US Food and Drug Administration for the treatment of exacerbations of MS in adults. RCI contains a naturally sourced complex mixture of purified adrenocorticotropic hormone (ACTH) analogs and other pituitary peptides (5). A trial comparing RCI vs. corticosteroids to treat MS exacerbations demonstrated similar, marked improvement in MS exacerbations with these drugs (6). In addition, RCI has demonstrated positive clinical outcomes and lower numbers of adverse events (AEs) than corticosteroids for patients in whom methylprednisolone treatment previously failed (7). The therapeutic benefits of RCI are often ascribed to endogenous corticosteroid production resulting from the activation of melanocortin receptor 2 (MC2R). However, recent evidence from preclinical studies suggests that RCI activates MC2R to a lesser extent than other ACTH formulations and, consequently, stimulates less endogenous corticosteroid production (8, 9). Additionally, the immunomodulatory and anti-inflammatory effects of RCI may be mediated through corticosteroid-independent mechanisms via engagement of MC1R, MC3R, MC4R, and MC5R on immune cells (8, 10–13).

Although the use of RCI in MS has increased over the last decade, there is limited information available regarding the relationship among patient demographics, disease characteristics, dosing regimens, and short- and long-term effects of intermittent RCI use for the treatment of MS relapses. The goal of this study was to describe treatment patterns, relapse recovery, and safety outcomes for patients receiving RCI for the treatment of acute MS relapse.

## Methods

### Study Design

**Figure 1:**
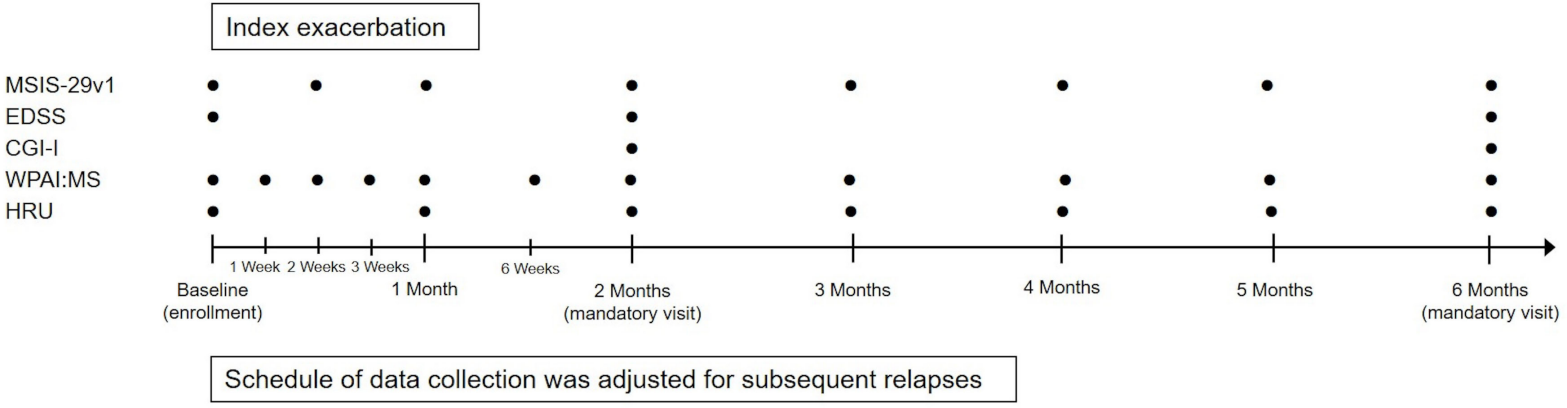
Study design and data collection. CGI-I, Clinical Global Impression-Improvement; EDSS, Expanded Disability Status Scale; HRU, Health Resource Utilization; MSIS-29v1, Multiple Sclerosis Impact Scale Version 1; WPAI:MS, Work Productivity and Activity Impairment Questionnaire: Multiple Sclerosis.

This was a multicenter, prospective, observational registry that enrolled patients with MS who were being treated with RCI for MS exacerbations across 31 neurology clinics in the US (Supplementary Table 1). All treatment decisions were made at the discretion of the patient's health care provider and were not mandated by the study design or protocol. Study design and an overview of data collection are presented in Figure 1.

The decision to initiate RCI occurred before determining whether the patient was interested and eligible to participate in the study. Patients deemed by the investigator to be appropriate for RCI treatment were entered into the study. Each enrolled patient was followed for up to 24 Months or until the earliest of study termination, patient withdrawal of consent, death, transfer to another facility, or 6 Months after the last patient was enrolled into the study.

The primary endpoint was change in MS Impact Scale Version 1 (MSIS-29v1) physical subscale score from baseline at Month 2. MSIS-29v1 is a validated, 29-item scale composed of 2 subscales that measure the physical (20 items) and psychological impact (nine items) of MS (14). Each item rates the extent to which MS impacts various aspects of day-to-day life; scores range from 1 (not at all) to 5 (extremely). Each item score is summed and converted to a 0–100 scale where 100 is the worst possible score. Patients were also assessed with the Expanded Disability Status Scale (EDSS), a clinical rating scale with scores that range from 0 (normal neurological examination) to 10 (death due to MS) (15); Clinical Global Impression of Improvement (CGI-I) scale, a clinician-rated scale that compares the change in the current global condition of the patient with their condition at baseline on a scale from 1 (very much improved) to 7 (very much worse) (16); the Health Resource Utilization (HRU) questionnaire, which assesses the number of MS-related doctor visits, health care professional home visits, emergency department visits, hospitalizations, paid and unpaid caregiving, and days of work missed for unpaid caregivers in the prior Month; and the Work Productivity and Activity Impairment Questionnaire: Multiple Sclerosis (WPAI:MS). The WPAI:MS measures absenteeism (work time missed), presenteeism (impairment at work), work productivity (overall work impairment), and activity impairment (17). WPAI:MS results are expressed as percentages, with higher percentages indicating greater impairment and less productivity.

Because it was possible for patients to have more than one exacerbation during the follow-up period, the exacerbation at the enrollment visit was defined as the index exacerbation and subsequent exacerbations were defined as relapses. The index exacerbation and subsequent relapses were defined as a new neurologic symptom(s) persisting for >24 h and accompanied by objective change(s) in neurological examination against a background of disease course stability over at least 1 Month before enrollment for the index exacerbation or relapse date. All relapses were confirmed via examination by the treating clinician as per usual care. Detailed data collection was undertaken for all relapses occurring during the study period.

Data were collected from patient medical charts and clinician assessments, and patient self-reports were undertaken at baseline and predefined time points during the follow-up study period after enrollment. Serious AEs (SAEs) and serious adverse drug reactions associated with the use of RCI were reported to the sponsor's pharmacovigilance department within 24 h of identification.

RCI was obtained through usual commercial channels for prescription medication. RCI was not provided free of charge by the sponsor. The patients' clinicians made all treatment decisions according to their usual practices and provided prescriptions for their patients, as appropriate. Clinicians made the decision to treat the patient before the decision to enter the patient into the study. There were no protocol-mandated medical procedures or diagnostic tests.

### Study Population

The target study population included adult patients (≥18 years of age) with a form of RRMS for whom the decision to initiate treatment of an MS exacerbation with RCI was made and who met the enrollment criteria and provided informed consent. Patients were recruited for the study from the routine flow of patients at each study site, as usual care would dictate. Patients deemed potentially eligible for the study by their treating clinician were invited to participate in the study during a routine care visit after it was determined that the patient would initiate RCI for the treatment of an MS exacerbation. Key inclusion and exclusion criteria are presented in Table 1.

**Table 1:** Inclusion and exclusion criteria.

**Key inclusion criteria**	**Key exclusion criteria**
• Capable of providing informed consent • Male or female ≥18 years of age • Patient has a clinically definite relapsing form of MS according to McDonald Criteria (2010 revision) • Patient with an acute MS exacerbation as determined by their treating clinician • Patient planning to initiate RCI for the treatment of an acute MS exacerbation	• Patients with a diagnosis of progressive MS • Patients who require concomitant corticosteroid therapy • Patients receiving experimental drug therapy • Patients with a history of scleroderma, systemic fungal infections, ocular herpes simplex, or cancer within prior 5 years • Patients who had recent surgery or have a history of or the presence of a peptic ulcer within 6 Months before study entry, congestive heart failure, or sensitivity to proteins of porcine origin • If female, pregnant or breast-feeding; or, if of childbearing age, an unwillingness to use appropriate birth control

*MS, multiple sclerosis; RCI, repository corticotropin injection*.

### Data Collection

The following information was collected at the enrollment visit: disease-modifying therapies (DMTs) and MS exacerbation-related treatments used within 2 years before index exacerbation; the number of prior MS exacerbations within 2 years before the index exacerbation; MS exacerbation symptoms within 2 years before the index exacerbation; time since the last MS exacerbation within 2 years before the index exacerbation; and the prescribed RCI regimen. In addition, the following scales were completed: the MSIS-29v1 (to evaluate the physical and psychological impact of MS), EDSS, WPAI:MS, and HRU. The MSIS-29v1 was also assessed remotely at 2 weeks post-baseline as well as monthly up to 6 Months post-baseline. Patients were asked to complete an electronic diary daily while on RCI to collect data on actual RCI use.

At each usual care visit during the 6-Month period after index exacerbation and any relapses, site staff collected data from patient medical records on DMTs, MS-related and other concomitant medications, magnetic resonance imaging received per usual care, AEs, and SAEs. At mandated visits at the study sites 2 Months (±2 weeks) and 6 Months (±1 Month) after index exacerbation and any relapses, clinicians completed the EDSS and CGI-I to assess exacerbation improvement. If a patient had one or more relapses following the index exacerbation, the schedule of follow-up assessments was restarted based on the timing of the latest relapse.

### Statistical Analyses

Effectiveness analyses were completed in the intention-to-treat (ITT) population, defined as all patients who received at least 1 dose of RCI and who contributed any data to the study. With an assumed change from baseline of 7 units (SD 19) for the MSIS-29v1 physical subscale score, a sample size of 80 patients was determined to provide 90% power to detect a nonzero change from baseline with a significance level of 0.05. Changes from baseline for MSIS-29v1, EDSS, WPAI:MS, and HRU assessments were summarized using descriptive statistics; tests of the null hypothesis that the mean change from baseline is equal to zero were carried out using 2-sided paired *t*-tests (or Wilcoxon signed rank test if the data were not normal). CGI-I *P*-values were based on the Wilcoxon signed rank test for the null hypothesis of no change (i.e., median score = 4). Outcomes that are defined as proportions were summarized using frequencies, percentages, and 2-sided 95% confidence intervals.

Safety analyses were completed for the safety population, defined as all patients who received at least 1 dose of RCI. The number of AEs and SAEs and the number of patients reporting AEs and SAEs were listed and summarized descriptively.

### Ethics

This study was conducted in accordance with International Council for Harmonization of Technical Requirements for Pharmaceuticals for Human Use Good Clinical Practice guidelines (as they apply to observational research), all applicable patient privacy requirements, and the ethical principles that are outlined in the Declaration of Helsinki 2008. The study was reviewed and approved by Advarra® (formerly Quorum Review IRB; Columbia, MD, USA).

## Results

**Figure 2:**
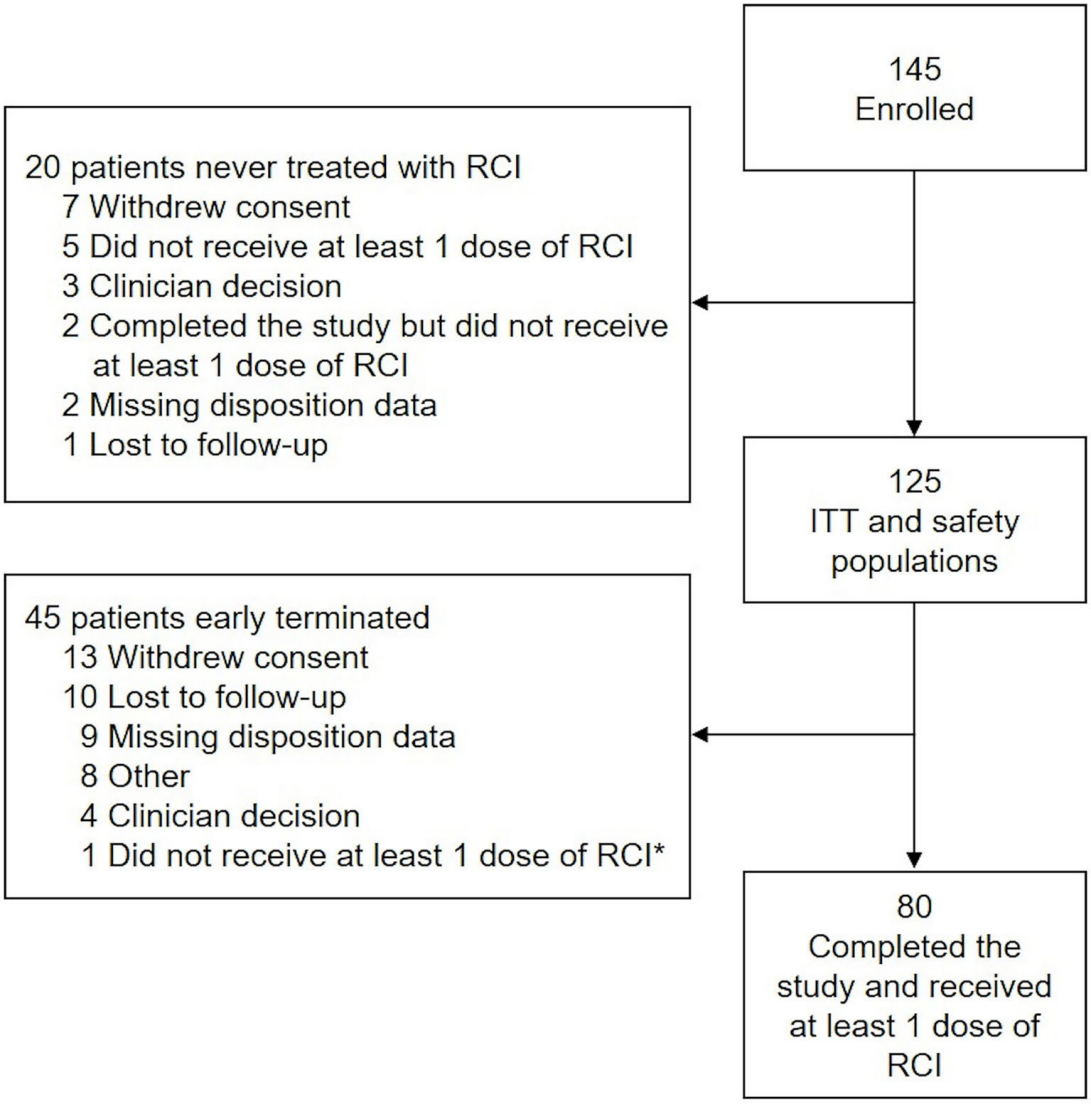
Study disposition and analysis. *Patient did receive at least 1 dose of RCI and was included in the ITT and safety populations. The disposition reason selected (i.e., “did not receive at least 1 dose of RCI”) was a data entry error. ITT, intention-to-treat; RCI, repository corticotropin injection.

**Figure 3:**
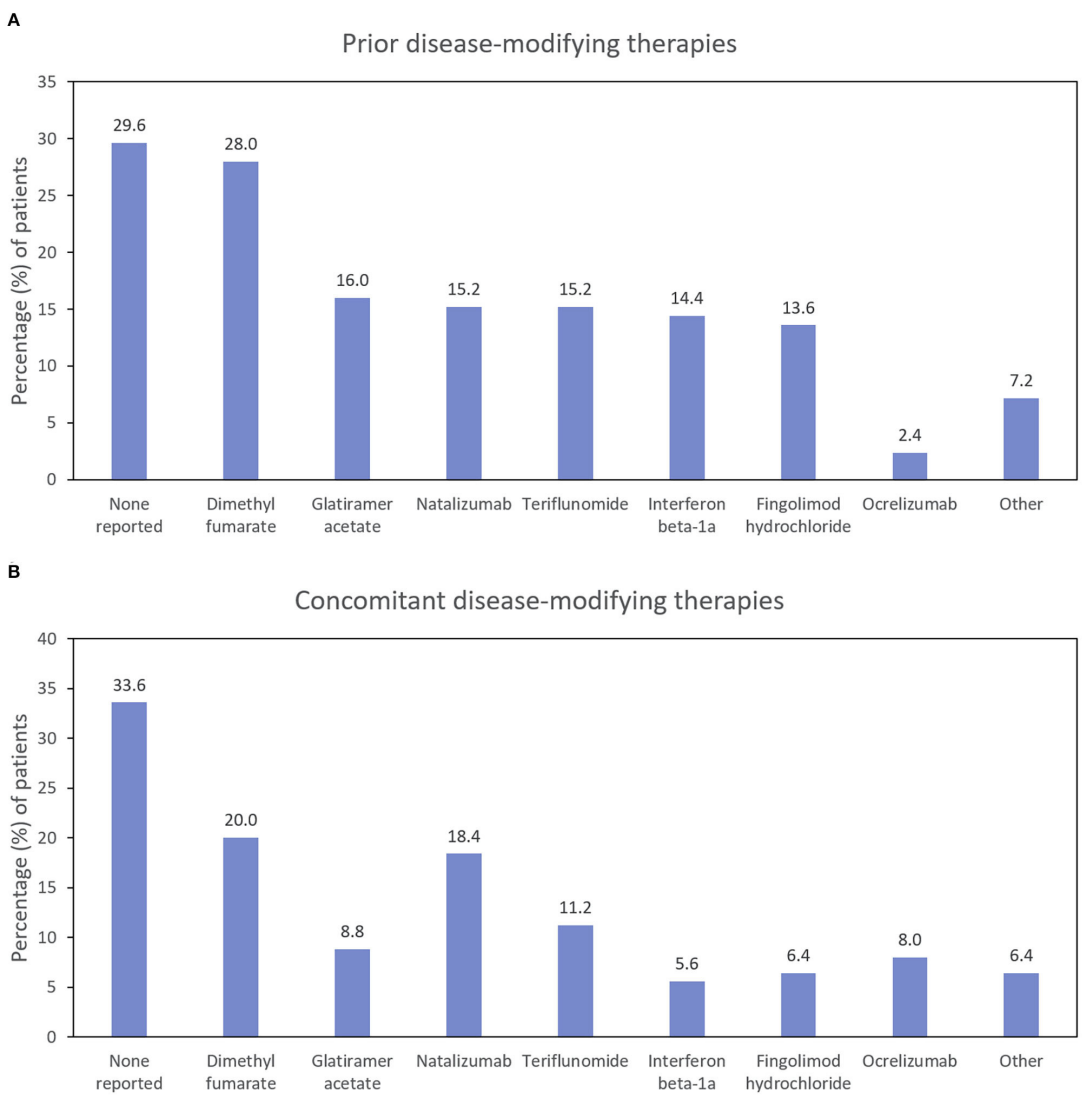
Percentage of patients using disease-modifying therapies before **(A)** and during **(B)** the study (ITT population; *N* = 125). ITT, intention-to-treat.

The first patient was enrolled on November 24, 2015, and the last patient completed the study on May 8, 2019. Of 145 patients enrolled, 82 (56.6%) completed 24 Months of follow-up. The ITT population comprised 125 patients; 80 patients completed the study and received at least 1 dose of RCI (Figure 2). Mean age was 47 years; 88.0% of patients were female, and 84.0% were Caucasian. The average time since diagnosis of MS was 10.2 years; 58.4% of patients had experienced a relapse within the last 2 years, and 60.0% had a history of insufficient treatment response, intolerance, or limited intravenous access associated with high-dose corticosteroids. Detailed demographic information, relapse history, and baseline assessment scores at the time of RCI initiation for an acute MS exacerbation are presented in Table 2. DMTs used during the previous 2 years as well as concomitant DMTs are presented in Figure 3.

**Table 2:** Demographics and baseline assessment scores.

**Characteristic**	***N***	**ITT Population *N* = 125**
Age (years), mean (SD)	124	47.0 (12.1)
Gender, *n* (%)	125	
Male		14 (11.2)
Female		110 (88.0)
Missing		1 (0.8)
Race, *n* (%)	125	
White		105 (84.0)
Black or African American		14 (11.2)
No information		3 (2.4)
Other		2 (1.6)
Missing		1 (0.8)
Time since initial MS diagnosis (years), mean (SD)	121	10.2 (8.3)
Hx of insufficient response, intolerance, or IV access issues with high dose CSs for an MS relapse, *n* (%)	125	75 (60.0)
Number of relapses within the last 2 years, *n* (%)	125	
0		50 (40.0)
1		55 (44.0)
2		11 (8.8)
3		7 (5.6)
Missing		2 (1.6)
Employment status, *n* (%)	125	
Employed full-time or part-time		50 (40.0)
Disabled		26 (20.8)
Retired or not working		35 (28.0)
Other		13 (10.4)
Missing		1 (0.8)
Efficacy measures, mean (SD)		
MSIS-29v1 physical subscale (range 0–100)	96	55.7 (24.4)
MSIS-29v1 psychological subscale (range 0–100)	96	57.2 (24.6)
EDSS (range 0–10)	108	3.9 (2.0)
WPAI:MS, mean (SD) (range 0–100)		
Percent work time missed due to MS	40	39.1 (38.4)
Percent impairment while working due to MS	41	45.1 (33.4)
Percent overall work impairment due to MS	40	29.7 (24.5)
Percent activity impairment due to MS	92	66.3 (25.3)
HRU questionnaire, mean (SD) (No. within the prior Month)	98	
All MS-related doctor's office or clinic visits		1.4 (1.4)
MS-related doctor's office or clinic visits with a specialist		1.1 (1.1)
MS-related doctor's office or clinic visits with a general practitioner		0.3 (0.6)
MS-related health care professional visits at home		0.2 (1.0)
MS-related ED visits		0.1 (0.4)
MS-related hospitalizations with overnight stays		0.1 (0.5)
MS-related days in hospital		0.1 (0.8)
Days of paid caregiver assistance received due to MS		0.6 (2.4)
Days of unpaid caregiver assistance received due to MS		6.3 (10.9)
Days unpaid caregiver missed work due to the patient's MS		0.5 (1.8)

*CS, corticosteroid; ED, emergency department; EDSS, Expanded Disability Status Scale; HRU, Health Resource Utilization; Hx, history; ITT; intention-to-treat; IV, intravenous; MS, multiple sclerosis; MSIS-29v1, Multiple Sclerosis Impact Scale Version 1; SD, standard deviation; WPAI:MS, Work Productivity and Activity Impairment Questionnaire: Multiple Sclerosis*.

**Figure 4:**
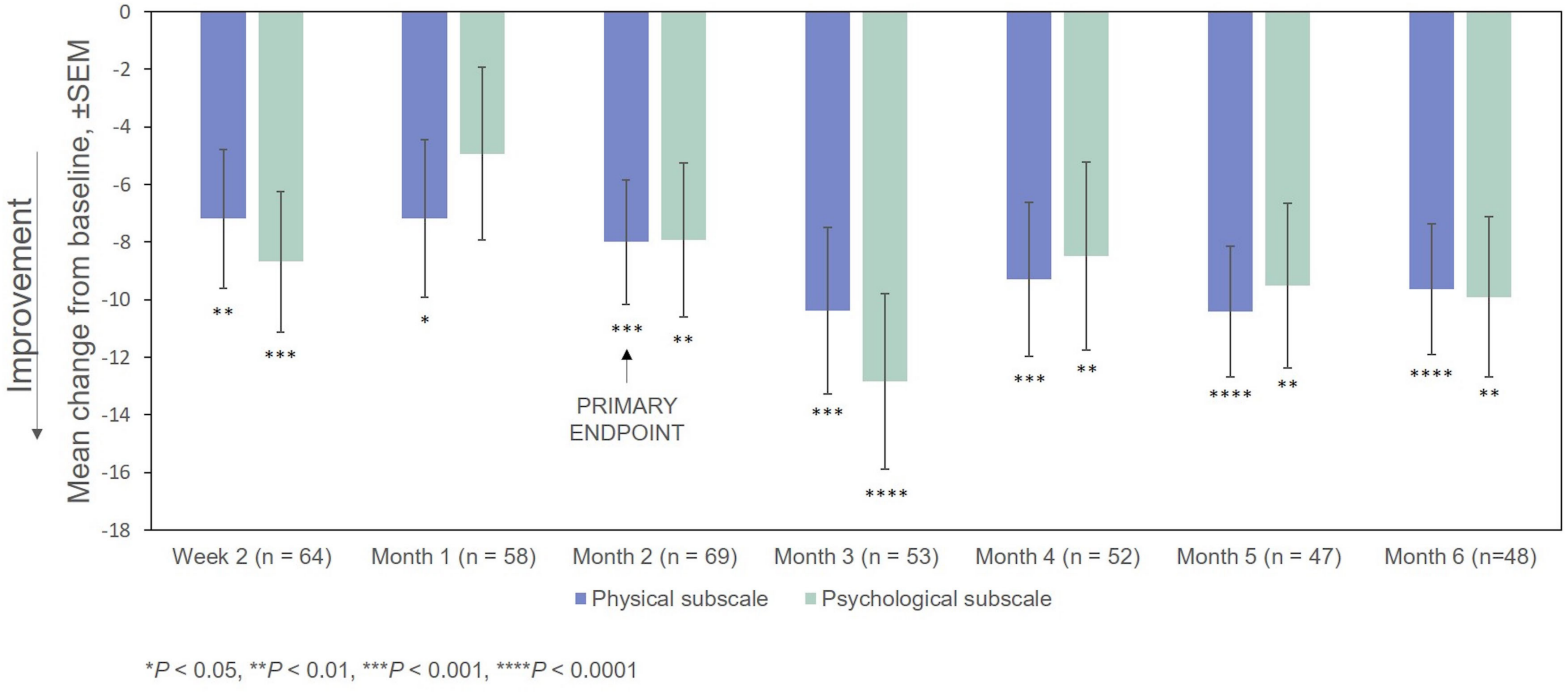
Mean change from baseline in the MSIS-29v1 scale. *P*-values are based on Wilcoxon signed rank tests (ITT population) compared with baseline. ITT, intention-to-treat; MSIS-29v1, Multiple Sclerosis Impact Scale Version 1; SEM, standard error of the mean.

**Figure 5:**
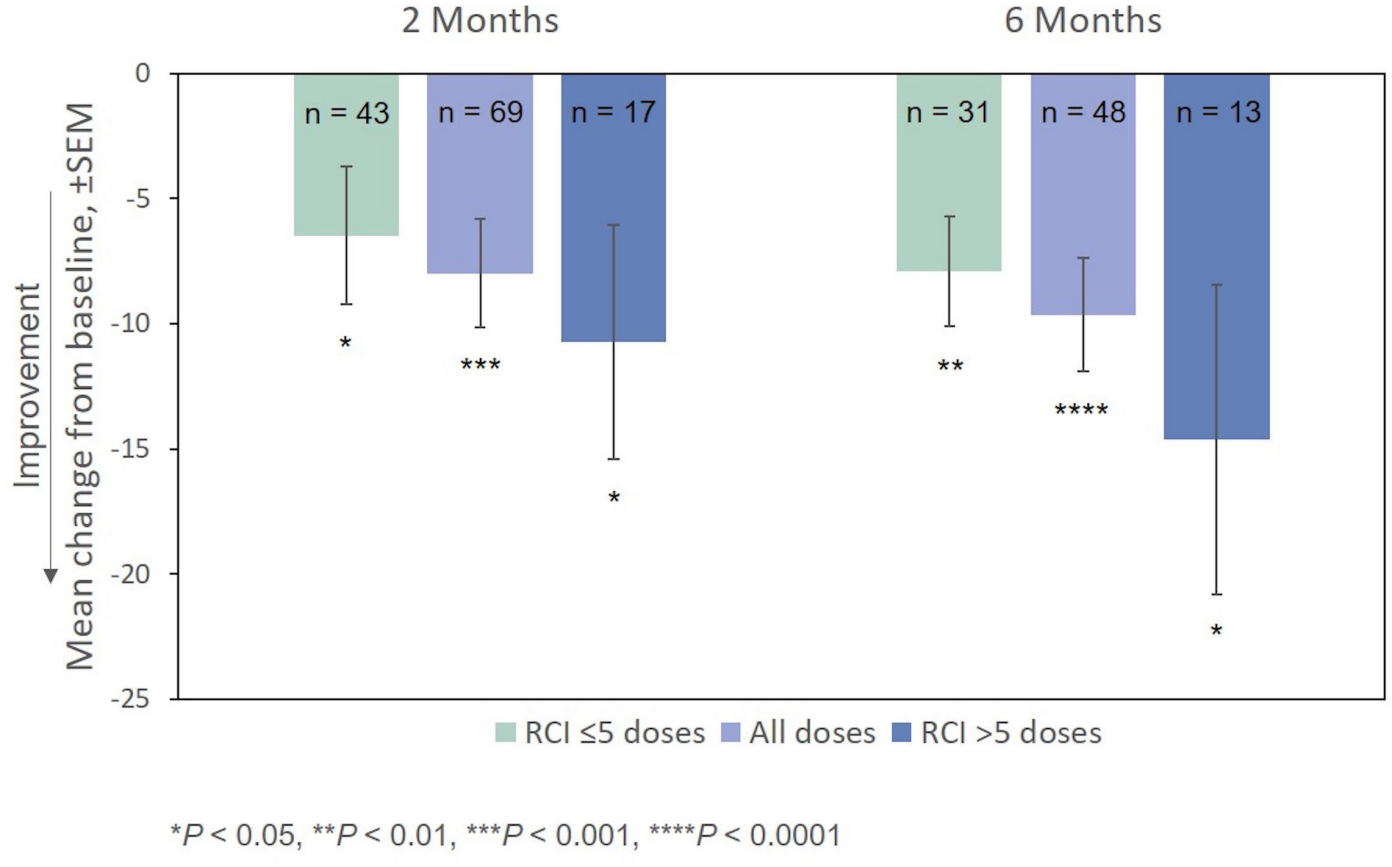
Mean change from baseline in the MSIS-29v1 physical subscale, by number of doses administered. *P*-values are based on Wilcoxon signed rank tests (ITT population) compared with baseline. ITT, intention-to-treat; MSIS-29v1, Multiple Sclerosis Impact Scale Version 1; RCI, repository corticotropin injection; SEM, standard error of the mean.

After treatment with RCI, mean MSIS-29v1 physical subscale scores decreased significantly at all time points post-baseline, including at Month 2, which was the primary endpoint. Scores also decreased significantly at all time points (except for Month 2) on the psychological subscale (Figure 4). The effect of the number of doses of RCI administered to the patient on MSIS-29v1 physical subscale scores (*post hoc* analysis) is presented in Figure 5. No direct statistical comparison was made, but there was a clear trend that patients who received >5 doses of RCI showed more improvement than patients who took ≤5 doses. Mean improvement in MSIS-29v1 physical subscale scores was statistically significant (vs. baseline) for both dose ranges.

**Figure 6:**
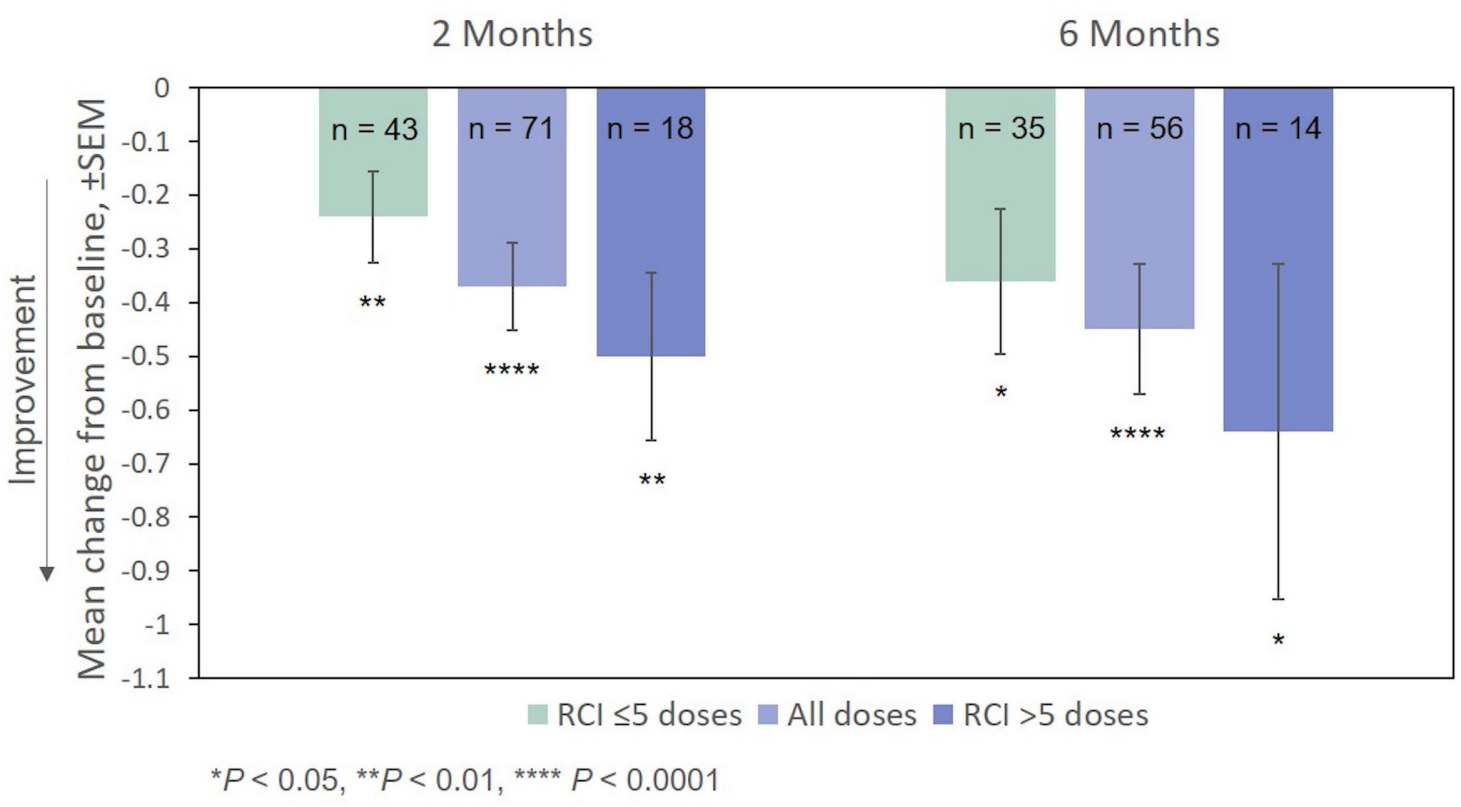
Mean change from baseline in the EDSS scale, by number of doses administered. *P*-values are based on Wilcoxon signed rank tests (ITT population) compared with baseline. EDSS, Expanded Disability Status Scale; ITT, intention-to-treat; RCI, repository corticotropin injection; SEM, standard error of the mean.

Improvement from baseline in EDSS scores at Months 2 and 6 was also statistically significant (Figure 6). The effect of the number of doses of RCI administered to patients on EDSS scores (*post hoc* analysis) is also presented in Figure 6. Again, no direct statistical comparison was made, but there was a clear trend of greater improvement observed in patients who received >5 doses of RCI. Mean improvement in EDSS scores was statistically significant (vs. baseline) for both dose ranges.

**Figure 7:**
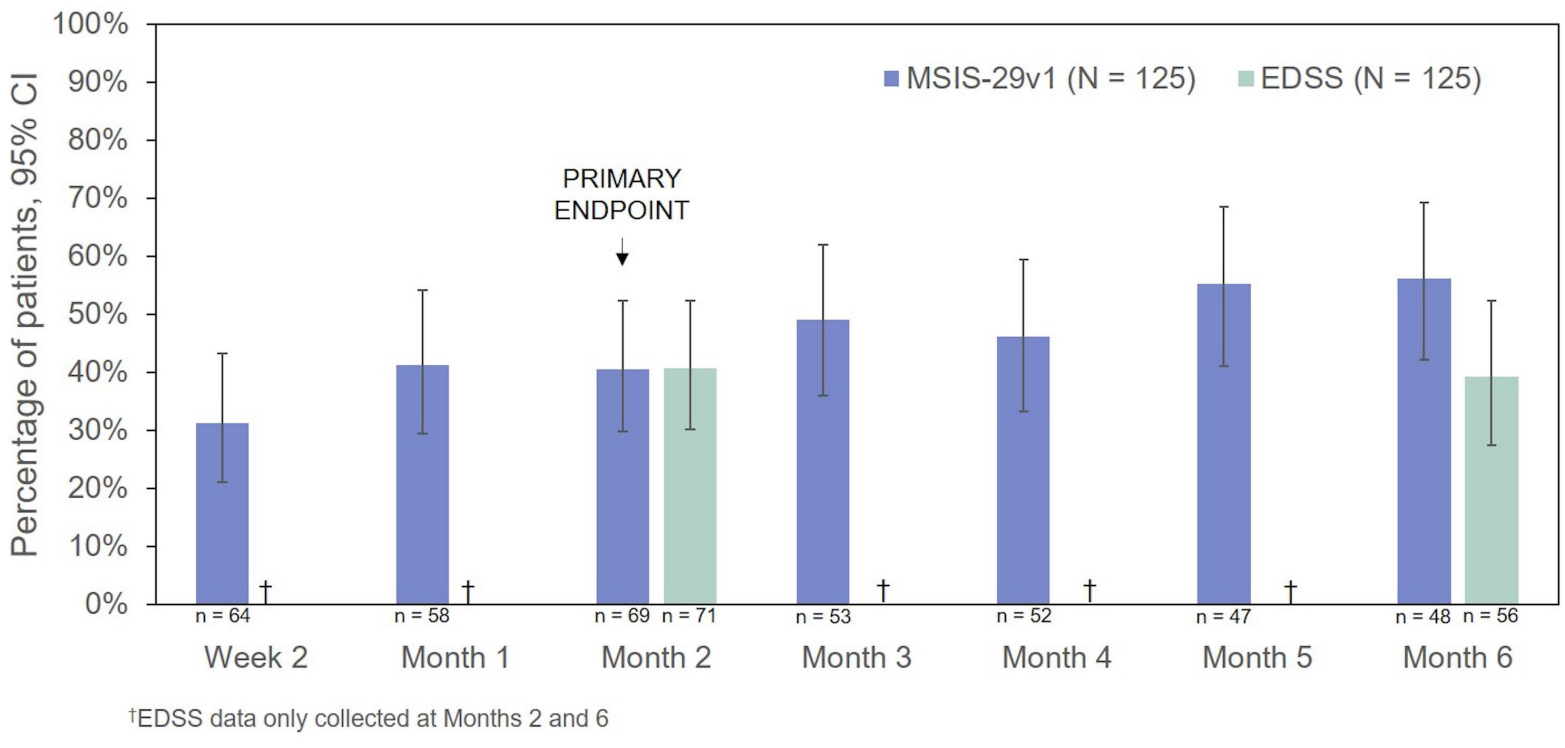
Percentage of patients who were responders to treatment (MSIS-29v1 physical subscale: ≥8 points improvement; EDSS: ≥0.5 points improvement). CI, confidence interval; EDSS, Expanded Disability Status Scale; MSIS-29v1, Multiple Sclerosis Impact Scale Version 1.

The percentages of RCI responders on the MSIS-29v1 physical subscale and EDSS are presented in Figure 7. On the basis of the MSIS-29v1 physical subscale, the percentage of responders increased over the course of the observation period from 31.3% (Week 2) to 56.3% (Month 6). The percentage of EDSS responders was 40.8% at 2 Months and decreased only slightly at 6 Months (39.3%). CGI-I scores indicated improvement in 63.4% of patients (45/71, *P* < 0.0001) at 2 Months and 61.4% of patients at 6 Months (35/57, *P* < 0.0001).

Approximately two-thirds of the patients reported using concomitant DMTs at some point during the study period (Figure 3). The proportion of patients taking natalizumab and ocrelizumab increased somewhat during the study (compared with the prior 2 years), and the proportion of patients taking glatiramer acetate, interferon beta-1a, and fingolimod hydrochloride decreased.

**Figure 8:**
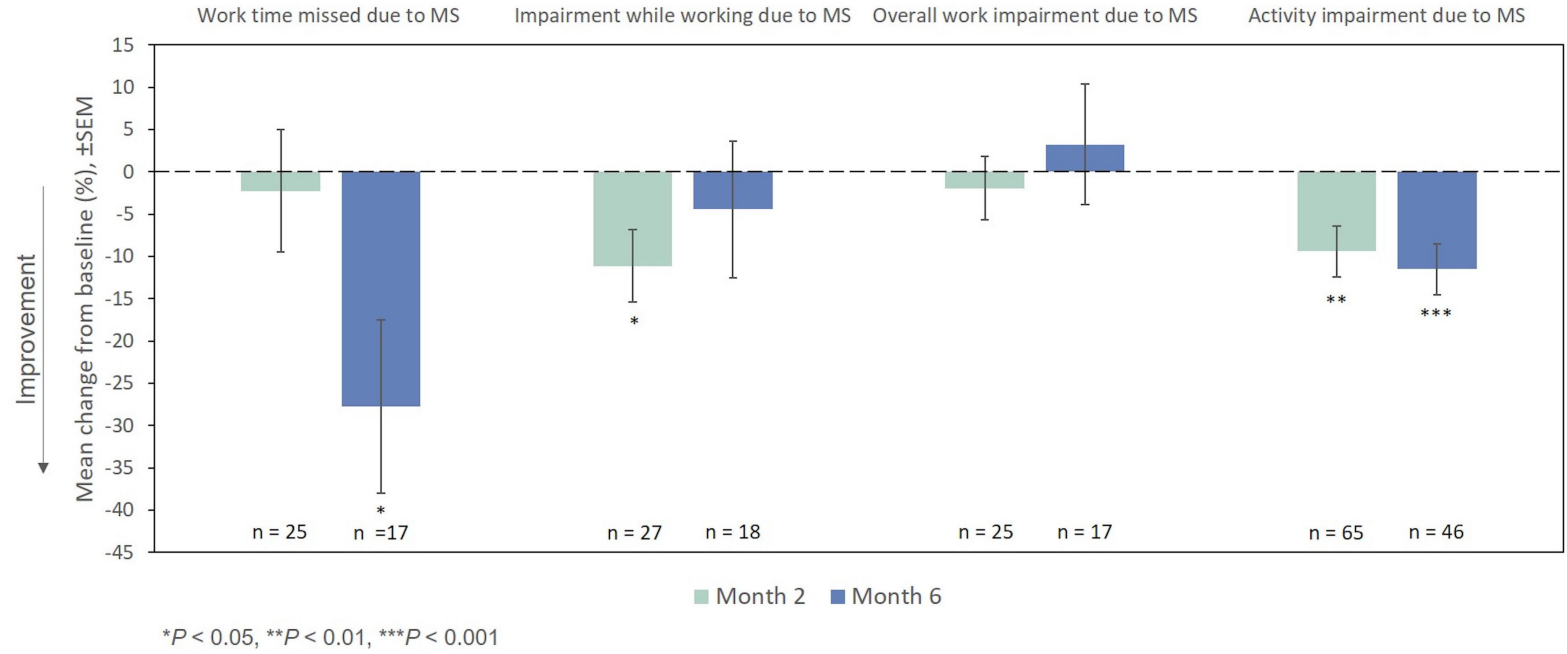
Mean change from baseline in WPAI:MS domains. *P*-values are based on Wilcoxon signed rank tests (ITT population) compared with baseline. ITT, intention-to-treat; MS, multiple sclerosis; SEM, standard error of the mean; WPAI:MS, Work Productivity and Activity Impairment Questionnaire: Multiple Sclerosis.

On the WPAI:MS activity impairment domain, significant improvements from baseline were observed at Months 2 and 6 (Figure 8). Among the small number of patients who were employed full-time or part-time at baseline (*n* = 50), changes from the index exacerbation on the absenteeism and presenteeism domains were significant at Months 6 and 2, respectively, while significant changes were not demonstrated at any time point for the lost work productivity domain after treatment with RCI.

**Figure 9:**
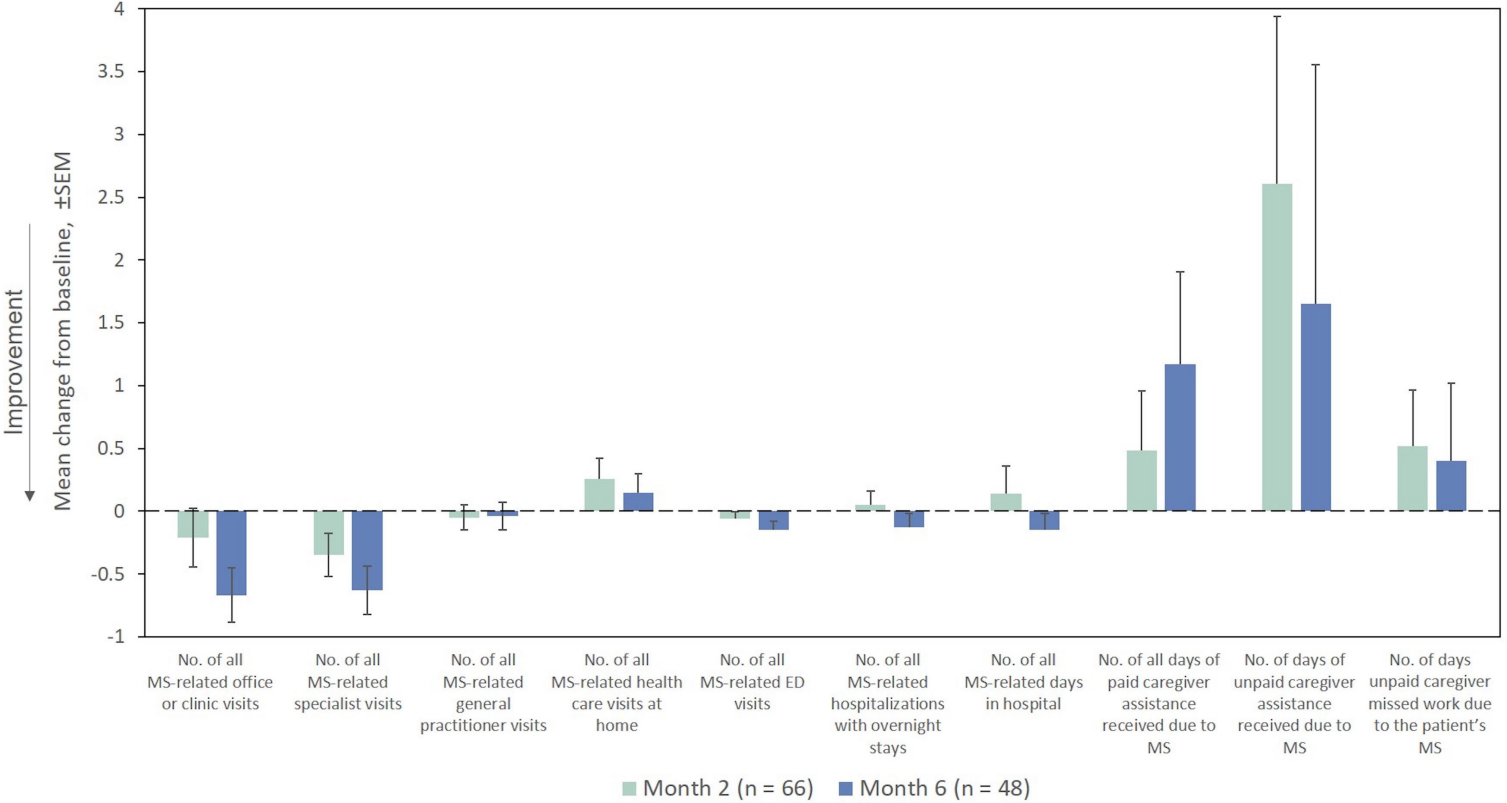
Mean change from baseline for HRU measures in the Month prior (ITT population). ED, emergency department; HRU, Health Resource Utilization; ITT, intention-to-treat; MS, multiple sclerosis; SEM, standard error of the mean.

The mean number of all MS-related outpatient, specialist, and emergency department visits in the prior Month as measured by the HRU questionnaire decreased from the index exacerbation at Months 2 and 6 (Figure 9). The remaining HRU measures did not demonstrate marked improvements from the index exacerbation after RCI treatment at any time point assessed.

A total of 83 AEs were reported by 35 (28.0%) patients. The most common AEs were MS relapse (4.0%), urinary tract infection (3.2%), peripheral edema (2.4%), nasopharyngitis (2.4%), cellulitis (1.6%), asthenia (1.6%), back pain (1.6%), dyspnea (1.6%), fall (1.6%), headache (1.6%), nausea (1.6%), and rash (1.6%) (Table 3). A total of 16 SAEs were reported by 11 (8.8%) patients. The most common SAEs were MS relapse (4.0%), asthenia (1.6%), and urinary tract infection (1.6%). No deaths were reported (Table 3).

**Table 3:** Summary of adverse events and serious adverse events, safety population (*N* = 125).

**Event**	**Patients with adverse events, *n* (%)**	**Number of events**
Adverse events^*^	35 (28.0)	83
Multiple sclerosis relapse	5 (4.0)	6
Urinary tract infection	4 (3.2)	4
Nasopharyngitis	3 (2.4)	3
Peripheral edema	3 (2.4)	3
Asthenia	2 (1.6)	2
Back pain	2 (1.6)	2
Cellulitis	2 (1.6)	2
Dyspnea	2 (1.6)	2
Fall	2 (1.6)	2
Headache	2 (1.6)	2
Nausea	2 (1.6)	2
Rash	2 (1.6)	2
**Serious adverse events**	11 (8.8)	16
Multiple sclerosis relapse	5 (4.0)	6
Urinary tract infection	2 (1.6)	2
Asthenia	2 (1.6)	2
Cellulitis	1 (0.8)	1
Concussion	1 (0.8)	1
Intentional overdose	1 (0.8)	1
Atrial fibrillation	1 (0.8)	1
Dehydration	1 (0.8)	1
Dyspnea	1 (0.8)	1

* *Adverse events occurring in ≥1.0% of patients*.

## Discussion

In this registry of patients with RRMS treated with RCI, participants showed significant improvement, compared with baseline, on the MSIS-29v1 physical subscale at 2 Months post-baseline (the primary endpoint). All other time points were statistically significant as well. Patients also showed statistically significant improvement on the MSIS-29v1 psychological subscale at all time points measured except Week 4. Clinician-reported outcomes (EDSS and CGI-I) were also significantly improved at Months 2 and 6.

Significant improvements on the WPAI:MS activity impairment domain and decreases in MS-related outpatient, specialist, and emergency department visits as reported in the HRU were noted with RCI therapy. MS has appreciable impact on patient functioning and work productivity. However, the impact of RCI on WPAI:MS work productivity measures in this study was unremarkable owing to the small number of patients who were employed full-time or part-time. Similarly, a lack of observed reductions in other HRU measures was likely a result of limited observations.

For both the MSIS-29v1 physical subscale and the EDSS, mean improvement from baseline was greater for patients who took >5 doses of RCI. Because of the small numbers of patients, these results need confirmation. However, even for patients who took ≤5 doses, improvement on these scales was statistically significant. The recommended dosing in the package insert for MS exacerbations is 14–21 daily doses (80–120 U) (5). Thus, the average patient in this study received only a fraction of the recommended dose of RCI.

As measured by the MSIS-29v1, the percentage of responders continued to rise over the course of the observation period. However, this was not the case for the EDSS, which remained essentially unchanged from Months 2 to 6. Although it is possible that the EDSS may be less sensitive in detecting change than the MSIS-29v1, as shown in Figure 6, average scores on the EDSS did vary with the number of RCI doses administered (Month 2: −0.2 to −0.5 points; Month 6: −0.4 to −0.6 points).

### Comparison With Corticosteroids

Several studies have investigated the use of corticosteroids for the treatment of MS relapse. For example, it was previously shown that treatment of MS relapse with methylprednisolone led to a response rate of 52% at 6 Months (a 1-point increase in the EDSS was used as the response criterion) (18). Similarly, Sellebjerg et al. (19) found that, after 1, 3, and 8 weeks of treatment with methylprednisolone, 31%, 54%, and 65% of patients, respectively, improved at least 1 point on the EDSS and had an average improvement of 1 point by 8 weeks. Milligan et al. (20) found improved EDSS scores (≥1 point) in 77% of MS patients with acute relapse after 4 weeks of treatment with methylprednisolone and an average improvement on the EDSS of approximately 2 points. Finally, Filipović et al. (21) found a mean decrease of 1 point on the EDSS after 5 days of treatment with methylprednisolone.

The observational nature of the present study may have contributed to the smaller improvements seen in the EDSS with RCI as compared with these earlier studies of corticosteroids. An earlier study found methylprednisolone treatment resulted in more rapid clinical improvement but was not superior to ACTH 3 Months after treatment (22). Also, Thompson et al. (6) compared methylprednisolone and ACTH and concluded there was no difference in the rate of recovery or final outcome.

The average ages of the patients in the four MS relapse studies using corticosteroids cited above were 34.0 (19), 36.9 (20), 37.5 (21), and 33.4 (22). The average age of the patients in this study was 47 years, with duration of MS averaging more than 10 years. There is evidence that age negatively impacts relapse recovery following treatment with corticosteroids (23, 24). In addition, patients in the present study were receiving more modern and effective DMTs than typical MS patients, with relatively few patients receiving older agents such as interferon beta-1a or glatiramer acetate, which suggests that these patients had more advanced disease. Thus, it is likely that these RCI-treated patients represent a subset of patients who are older and whose MS is more difficult to treat than most patients with MS.

No new safety signals for RCI were identified in this observational registry study. Notably, patients reported a low incidence (<1%) of RCI-related neuropsychiatric symptoms (e.g., changes in mood or behavior). Conversely, corticosteroids are associated with AEs that may mimic or exacerbate neuropsychiatric symptoms experienced during MS relapse (25). Further, a previous retrospective evaluation of case studies suggested that RCI exhibits a lower incidence of neuropsychiatric AEs than corticosteroids and, therefore, may be a preferred alternative to corticosteroids for select patients (25). However, this warrants further investigation in a prospective comparison of AEs in patients who have received treatment for MS relapse with RCI or a corticosteroid.

### Strengths and Limitations

The main strength of registries is that they reflect real-world evidence of treatment, and for this reason they are becoming more popular. However, that strength brings with it an important limitation that is not found in randomized clinical trials. Because registries are observational, there is no active comparator or placebo against which to measure efficacy (or randomization to treatments). Therefore, one cannot determine if improvement in patient outcomes is the result of the treatment being studied. Though one can track efficacy from baseline through the weeks and Months that follow, it is not possible to determine what portion of any change in scores is caused by the treatment. Additionally, only about half of patients (56.6%) who were enrolled in the registry completed 24 Months of follow-up. However, the primary endpoint was assessed before the time of study completion (at 2 Months after the index exacerbation) and was evaluated in a larger percentage of patients (71.9%) than the percentage who completed the study.

A potential drawback in a naturalistic study is that the knowledge that one is being monitored may impact one's actions. However, it was emphasized in site training activities that the study protocol was not to interfere with usual care and treatment of patients and that a critical review of clinician practice was not an objective of the study.

## Conclusions

Results from this prospective observational study of RCI in patients with MS show clinically meaningful improvement in MSIS-29v1 physical subscale scores and MS-related outpatient, specialist, and emergency department visits, as well as statistically significant improvements in the MSIS-29v1 psychological subscale, clinician-rated scales (EDSS and CGI-I), and the WPAI:MS activity impairment domain. *Post hoc* analyses suggest that the number of doses can impact efficacy. These results support the efficacy of RCI as a treatment for MS relapse in patients who cannot tolerate corticosteroids or have an inadequate response to treatment with corticosteroids. Further, AEs and SAEs were consistent with the known safety profile of RCI.

## Data Availability Statement

Discussion of statistical endpoints and analysis are included in the article. Summary aggregate (basic) results (including adverse events information) and the study protocol will be available on clinicaltrials.gov (NCT02633033) when required by regulation. Individual de-identified patient data will not be disclosed. Requests for additional information should be directed to the company at medinfo@mnk.com.

## Ethics Statement

The studies involving human participants were reviewed and approved by Advarra® (formerly Quorum Review IRB; Columbia, MD, USA). The patients/participants provided their written informed consent to participate in this study.

## Author Contributions

JK, TM, and MB contributed to the collection and interpretation of study data and to review and editing of the manuscript. BD contributed to study conceptualization and interpretation of results and to review and editing of the manuscript. EZ conducted the statistical analysis and contributed to review and editing of the manuscript. All authors contributed to the article and approved the submitted version.

## Conflict of Interest

JK has received speaking fees from Amgen, Allergan, Alexion, Biogen, EMD Serono Lilly, Lundbeck, Mallinckrodt Pharmaceuticals, Sanofi-Genzyme, Teva, and Viela. TM is employed by Advanced Neurology of Colorado, LLC, and has received speaking and/or consulting fees from Acorda, Allergan, Amgen, Biogen, Genentech, Mallinckrodt, Novartis, Reven, Sanofi-Genzyme, and Teva and research support from Allergan, Adamas, Biogen, Elan, EMD Serono, Genentech, Ipsen, Mallinckrodt, Novartis, ONO, Sanofi-Genzyme, Sun Pharma, and Teva. MB is employed by Collier Neurologic Specialists, LLC, and has received honoraria and consulting fees from Acorda, Alexion, Avanir, Biogen, Celgene, Genentech, Mallinckrodt, Sanofi-Genzyme, and Teva. BD is a former employee of Mallinckrodt Pharmaceuticals. EZ is an employee of Mallinckrodt Pharmaceuticals. Acthar R. Gel is a registered trademark of a Mallinckrodt company. The authors declare that this study received funding from Mallinckrodt Pharmaceuticals. The funder was involved in the study design, collection, analysis, interpretation of data, writing of this article, and the decision to submit it for publication. The reviewer SR declared a past co-authorship with the authors to the handling editor.

## Abbreviations

ACTH: adrenocorticotropic hormone
AE: adverse event
CGI-I: Clinical Global Impression of Improvement
DMT: disease-modifying therapy
EDSS: Expanded Disability Status Scale
HRU: Health Resource Utilization
ITT: intention-to-treat
MCR: melanocortin receptor
MS: multiple sclerosis
MSIS-29v1: Multiple Sclerosis Impact Scale Version 1
RCI: repository corticotropin injection
RRMS: relapsing-remitting multiple sclerosis
SAE: serious adverse event
WPAI:MS: Work Productivity and Activity Impairment Questionnaire: Multiple Sclerosis.
