# Supplementary material for: A Prospective Observational Registry of Repository Corticotropin Injection (Acthar® Gel) for the Treatment of Multiple Sclerosis Relapse
Source: Front Neurol. 2020 Dec 22;11:598496. doi: 10.3389/fneur.2020.598496 (PMC7783159; doi:10.3389/fneur.2020.598496)
Supplement: Supplementary file 1 [file Data_Sheet_1.docx]

**Supplementary Material**

A Prospective Observational Registry of Repository Corticotropin Injection (Acthar^®^ Gel) for the Treatment of Multiple Sclerosis Relapse

Jeffrey Kaplan^1^, Tamara Miller^2^, Matthew Baker^3^, Bryan Due^4^, Enxu Zhao^4^

^1^Kansas City Multiple Sclerosis and Headache Center, Overland Park, KS, USA; ^2^Advanced Neurology of Colorado, LLC, Fort Collins, CO, USA; ^3^Collier Neurologic Specialists, LLC, Naples, FL, USA; ^4^Mallinckrodt Pharmaceuticals, Bedminster, NJ, USA

**Supplementary Table 1.** List of study sites

| **Name** | **Location** |
| --- | --- |
| Advanced Neurosciences Research, LLC | Fort Collins, CO |
| Alabama Neurology Associates | Homewood, AL |
| Alpha Neurology, PC | Staten Island, NY |
| Associated Neurologists of Southern Connecticut, PC | Fairfield, CT |
| Bon Secours Health System - Virginia | Richmond, VA |
| Braunstein Neurology | Mooresville, NC |
| College Park Family Care Center | Overland Park, KS |
| Collier Neurologic Specialists, LLC | Naples, FL |
| Columbus Research & Wellness Institute, Inc | Columbus, GA |
| Colonial Healthcare | Sumter, SC |
| Colorado Springs Neurological Associates | Colorado Springs, CO |
| Cordova Research Institute, LLC | Miami, FL |
| Desert Neurology Associates, LLC | Las Vegas, NV |
| Five Towns Neurology, PC | Woodmere, NY |
| Florida Neurological Center | Ocala, FL |
| International Neurorehabilitation Institute | Lutherville, MD |
| Hope Neurology | Knoxville, TN |
| Irene Greenhouse MD, PC | Jamison, PA |
| MedStar Health | Washington, DC |
| Meridian Clinical Research | Savannah, GA |
| Milford Regional Medical Center | Hopedale, MA |
| Neurological Services of Orlando, PA | Orlando, FL |
| Neurology and Stroke Associates | Lititz, PA |
| Neurology Associates of Ormond Beach | Ormond Beach, FL |
| Neurology Associates, PA | Maitland, FL |
| Neurology Center of New England, PC | Foxboro, MA |
| Oak Clinic for Multiple Sclerosis | Uniontown, OH |
| Ogden Clinic | Ogden, UT |
| Strotira, Inc. | Bethpage, NY |
| The Toledo Clinic | Toledo, OH |
| University System of Maryland | Baltimore, MD |
